# Translating lessons to reinforce national stillbirth response; multi-stakeholder perspectives regarding priorities and opportunities to deliver quality evidence-based interventions within a limited-resource context in Uganda

**DOI:** 10.1186/s12913-024-11180-z

**Published:** 2024-06-10

**Authors:** Eric Ssegujja, Michelle Andipatin

**Affiliations:** 1 Department of Health Policy Planning and Management, School of Public Health, College of Health Sciences, Makerere University, P.O. Box 7076, Kampala, Uganda; 2 School of Public Health, Faculty of Community and Health Sciences, University of the Western Cape, Cape Town, Republic of South Africa; 3 Department of Psychology, University of the Western Cape, Cape Town, South Africa

**Keywords:** Stillbirth, National-level strategies, Implementation context, Policy adaptation, Implementation challenges

## Abstract

**Background:**

There is noted increase in attention towards implementation of evidence-based interventions in response to the stillbirth burden in low- and middle-income countries including Uganda. Recent results reporting some of the strategies adopted have tended to focus much attention towards their overall effect on the stillbirth burden. More is needed regarding stakeholder reflections on priorities and opportunities for delivering quality services within a limited resource setting like Uganda. This paper bridges this knowledge gap.

**Methods:**

Data collection occurred between March and June 2019 at the national level. Qualitative interviews were analysed using a thematic analysis technique.

**Results:**

Identified priorities included; a focus on supportive functions such as the referral system, attention to the demand side component of maternal health services, and improvements in the support supervision particularly focusing on empowering subnational level actors. The need to strengthen the learning for better implementation of strategies which are compatible with context was also reported. A comprehensive and favourable policy environment with the potential to direct implementation of strategies, harnessing the private sector contribution as well as the role of national level champions and patient advocates to amplify national stillbirth reduction efforts for continued visibility and impact were recommended.

**Conclusion:**

Great potential exists within the current strategies to address the national stillbirth burden. However, priorities such as improving the supportive functions of MCH service delivery and attention to the demand side need to be pursued more for better service delivery with opportunities including a favourable policy environment primed to better serve the current strategies. This calls for dedicated efforts targeted at addressing gaps within the existing priorities and opportunities for better delivery of national strategies to address the stillbirth burden in Uganda.

**Supplementary Information:**

The online version contains supplementary material available at 10.1186/s12913-024-11180-z.

## Introduction

Globally, over two million stillbirths occur every year. Responding to the stillbirth burden is a key health system priority, especially in low- and middle-income countries which shoulder the highest burden including Uganda [1–3]. A chronology of events from the renewed global attention to the problem resulting in raising funds globally has contributed to the country’s prioritisation of evidence-based interventions that have now become embedded in national policies at least on paper and to some extent reflected in implementation practices in Uganda [2, 4, 5]. The attention became even more pronounced when the country established mechanisms for strengthening health systems, monitoring progress and evaluating intervention effectiveness. However, whereas strategies focusing on the mother and her unborn babies have been proven effective with relative resource availability, their effect following introduction into resource-limited health systems requires more amplification owing to variations in these and other implementation contexts.

The introduction of evidence-based health systems strengthening interventions has implications for the performance of the health systems in terms of resources and efficacy of such strategies in responding to the given public health challenge [6–8]. By 2021, the country was reported to experience a stillbirth burden of 17.8 stillbirths per 1,000 deliveries. Uganda is currently implementing several interventions to respond to the stillbirth burden along the Sexual and Reproductive Maternal Newborn and Child Health (SRMNCH) continuum of care [9]. Like in other limited resource health systems settings, there are variations in the health system’s responses when different strategies are rolled out as a component of the routine standard of care service provision hence presenting major challenges to their ability and effectiveness in responding to the given problem [10].

Evidence from elsewhere has demonstrated the important role that national-level key stakeholders play in facilitating the acceptability, adaptability and sustainability of innovations once introduced into the health systems [11–13]. Their influence may likely be crucial in the successful implementation of strategies to address the stillbirth burden in Uganda. MCH policy and implementation adjustments to accommodate the rolling out of evidence-based interventions into the health systems especially after 2011 prioritised both the supply side (health systems strengthening) and demand side (social determinants of health) [2, 8]. Input from national-level health systems managers and policymakers within the MCH policy networks remains very vital in providing insights and feedback for the successful implementation of health systems strengthening strategies to address the national stillbirth burden [14–16].

There is a growing need to assess the effectiveness of these evidence-based interventions such as data quality improvements and maternal and perinatal death reviews among others in supporting response to the stillbirth burden [17]. Perspectives from stakeholders have long been valued as a crucial component of the feedback loop for health systems including such platforms as joint review missions [18, 19]. Moreover, health systems strengthening efforts responding to stillbirth burden would benefit more from such feedback mechanism and though ideal, may require timely assessment with the possibility of ploughing such feedback into the re-design and adjustment of these approaches for better quality service delivery [2, 6, 7]. To respond to this need, we sought to explore the health systems managers’ and policymakers’ perspectives on priorities and opportunities to address the stillbirth burden in Uganda. This analysis, therefore, sought to answer the question; what are the key priorities and opportunities to deliver quality evidence-based interventions in Uganda according to multiple stakeholders? Insights into their perspectives is key as they are likely to shape the implementation of health systems strengthening approaches with feedback having the potential to inform revisions to the current practices.

## Methodology

### Study design and setting

To deepen the knowledge and understanding of national-level perspectives regarding strategies to address stillbirth in Uganda, in-depth, descriptive data was required. The qualitative methodology was preferred for this objective [20, 21]. Due to variability in individual worldviews and different subjective interpretations according to respondents’ categories, an exploratory approach was deemed the best-suited method of a qualitative inquiry [22]. This approach was both deductive and inductive which required the researcher to remain theoretically guided by the research question while at the same time being able to contextualise, make sense and interpret the data. The ability to navigate the double terrain allowed the researcher to explore the convergences and variations within the data in a systematic way. Details of this methodology have previously been described elsewhere [5].

The study was conducted in Uganda among national-level key informants drawn from the existing maternal and child health policy networks. It was conducted between March 1st and June 30th 2019 at the national level. At the time, the country had adopted and translated several recommended interventions to address the stillbirth burden into the health systems. Therefore, within this context, the results remain relevant at the time of publishing this paper. National stewardship was provided by the Ministry of Health at the centre with delegated responsibilities at the subnational level spearheaded by the Department of Health at the district level. The health system is organised along a tiered structure with the lowest level being the community structure commonly known as the Village Health Team (VHT) structure. This provides health promotion and prevention activities with members identified from within the community they live to offer these services voluntarily. Above the VHT is HCII; the first level that provided clinical outpatient services, immunisation, and family planning among others followed by HCIII which provides maternity services, inpatient care, basic laboratory services and minor surgery on top of what is provided at the HCII. The HCIV provides Comprehensive Emergency Obstetric Care (CEmOC) services, a standby ambulance, and centralised data collection together with what was provided at HCIII. The General Hospital offers plain X-ray examination, all general medical and surgical conditions as well as specialist services on top of what is provided at HCIV while the Regional Referral Hospital (RRH) offers all specialised services at the National Referral Hospital (NRH) save for its location at the regional level and absence of specialised institutes which are only located at the NRH such as the Uganda Heart Institute, Uganda Cancer Institute among others. The private sector bears the same replica in terms of service level organisation and it was comprised of the Private-Not-For-Profit (PNFP) and Private-For-Profit (PFP) with the former benefiting from some form of public funding while the latter’s main support from the government was data systems support, regulation and was mainly supervised through delegated authority to professional and compliance bodies. According to statistics, data reporting on the national stillbirth burden was predominantly from the public and PNFP facilities despite the contribution of the private sector.

### Study population and sample

The study population comprised respondents drawn from the national-level MCH policy networks. A maximum variation purposive sampling technique was employed. Potential respondents were pre-identified as eligible with guidance from individuals familiar with national-level MCH programming and policy processes. These were drawn from the national level MCH policy networks consisting of policymakers, academia, civil society representatives, donor community, researchers, Non-Governmental Organisations (NGO) representatives, Private sector players, and health systems managers among others. A list was generated with the contacts from which they were approached. Additional respondents were selected based on leads from the Ministry of Health and information from reports detailing their contribution to national-level policy processes. We employed a process tracking methodology together with purposive sampling to identify key documents for review.

### Data collection procedures

Initial registration of the study was obtained on the 13th of November 2017 from the Biomedical Research and Ethics Committee of the University of the Western Cape. Data collection was conducted between March 1st and June 30th 2019. Respondents were approached through telephone calls where the aims and objectives of the study were explained. It consisted of key informant interviews. The exercise was conducted by the first author with support from two graduate-level research assistants experienced in qualitative methodologies. On the day of the interviews, individual informed consent was obtained from all respondents before interviewing at the respondents’ places of work lasting from 45 min to one hour depending on the point of saturation. A flexible topic guide consisting of open-ended questions developed for this study was used (Supplementary material 1) [5, 23]. The topic guide explored respondent’s views regarding the national stillbirth reduction strategies and the implementation context with prompts used to aid recall in cases where information gaps manifested among others. Two callbacks were made in case the respondent was not available during the first attempt after which a replacement would be identified. On the day of the interview, a safe and secure place was identified from where the interview was conducted. Field notes were taken during the interview and at the end of each field day during the daily debrief meeting attended by the data collection team. Overall 20 out of the 23 respondents who had earlier been contacted took part in this study. At the sampling stage, maximum variance to include diverse respondents was ensured while at the analysis stage, the iterative data analysis across respondents’ categories was aimed at attaining data saturation. The three who had earlier expressed willingness to participate could not be accessed after the second callback. 17/20 were female and drawn from Professional Associations (6), Ministry of Health (5), NGOs (4), Private not for Profit (2), Academia/Researchers (2) and Private for Profit (1).

### Data management and analysis

All interviews were transcribed using Microsoft Office (MS) Word by two research assistants who had participated in the data collection. All transcripts were read by the author before exporting them to Atlas. ti a qualitative data management software for coding [24]. Analytical diaries were generated to record early reflections and ideas. The transcripts were then annotated with exploratory comments containing contextual and subjective information. Emergent themes were then reduced into codes which were followed by searching for connections within those themes. A deductive coding approach was adopted to assign text to their respective codes [25]. Text relating to a particular code was identified from the transcripts, highlighted and assigned as meaningful units to the respective codes. Thereafter query reports were run for each of the codes using a query tool function within the Atlas. ti software [26]. A manual pile sorting exercise was conducted for each of the queries by reading through each of the texts to identify underlying meanings. Texts with similar or related meanings were then grouped into separate piles. Each pile was then read through once more to identify underlying themes and sub-themes. The themes were identified iteratively and refined using a constant comparative, reflection, refinement and analysis of emerging themes [27]. It involved engaging with the theory, and data across respondents’ categories to achieve consensus in interpretation which allowed for nuances to emerge through the analytical process [26]. We then adopted the Consolidated Criteria for Reporting Qualitative Results (COREQ) checklist to guide the reporting of results. Triangulation was done through a comparison of results from the different respondents’ categories. Typical quotes have been reproduced within the results to bring out respondents’ voices. Particular pseudonyms reflecting respondents’ category at the time of data collection, the level of service provision and current role have been used to enhance anonymity and confidentiality.

## Results

Four major themes emerged as priorities by the respondents and they include; (1) Supportive functions for MCH service delivery, (2) Attention to the service user concerns, (3) Supervision improvement and (4) Strengthening of the learning component. Regarding opportunities, the themes which emerged were; (1) the favourable policy environment, (2) the harnessing of the private sector, (3) the role of national-level champions, and (4) a consolidation of global support. They are presented in Table 1 below;

**Table 1:** Summary of key findings

	Priorities	Opportunities
1	Supportive/ Supervision functions for MCH services • Referral systems strengthening • MCH diagnostics at HCIV • Governance and leadership • Strengthen regional structures to support supervision functions • Guideline/policy adherence,	Favourable policy environment • Uniform policy implementation • Opportunities for scaling up • Increased focus on newborn health.
2	Addressing patient-level concerns. • Quality concerns by service users and Provision of feedback to mothers • Community ownership and Political mobilisation for service uptake and interest.	Harness private sector contribution • Focus on urban midwifery practitioners and entrepreneurs • Involvement in quality improvement interventions
3	Improvement • Mechanisms to incorporate lessons learnt • Standardise lessons across the board • Promotion of data ownership, • Pre-service training on the application of evidence-based tools.	National level champions • Maintain the stillbirth agenda as a national priority. • Amplify patient advocates voices
		Consolidation of global support • The careful transition from donor to central support • Harmonisation of different donor support. • Proper use of resources from donors.

### Priorities

Reflecting on progress towards implementation of interventions to address stillbirths in Uganda, four priorities were synthesised from stakeholder responses; (1) supportive functions to the MCH service delivery, (2) attention to patient-level (service user) concerns, (3) improvements in supervision, and (4) strengthening the learning component. A distillation of each follows next with representative quotes;

### Supportive/supervision functions for effective MCH service delivery

Stakeholders viewed the supportive functions for effective MCH service delivery as of paramount importance. Supportive functions such as the referral systems, availability of fuel and a reliable source of power to run the operating theatres at Health Centre (HC) IVs were some of the aspects mentioned. They noted that these would affect the effectiveness of the available facility-based MCH services. The referral system contributed to delays and effecting community referrals was still a major challenge. The same was said of the difficulties experienced when patients were recommended for referrals. Whereas the policy on Comprehensive Emergency Obstetric Neonatal and Intensive Care (CEmONIC) was stipulated, making it beyond project mode responsive was highly dependent on the effectiveness of the referral system. HCIVs with functional theatres would sometimes experience fuel shortages which occasioned further delays or unnecessary referrals. Some HCIIIs are oftentimes faced with the unavailability of ambulatory services to call upon or lack of fuel to effect timely referrals. This was further compounded by the poor status of the road networks in some areas, especially in rural localities.

*So, there is a lot to be done in strengthening the emergency obstetric care services so that we can reduce the stillbirth rates in this country**** NLI008***

Respondents acknowledged that despite the operationalisation of CEmONIC at HCIVs, MCH service-related diagnostics did not reflect the same. Within the staffing norms and policy, there was a lack of provision for a sonographer to conduct ultrasound scans for mothers during Antenatal Care (ANC), a key service delivery gap. Paying attention to critical cadres such as midwives and anaesthetists was equally a priority worth considering by respondents. Some respondents reported that this would go a long way in supporting core MCH service activities such as the identification of potential risks to the foetus such as foetal growth restriction at that level which occasions unnecessary delays in decision-making due to the referrals out for this service.

*if the moms ultimately end up in the hospitals and the hospitals are not prepared for them because a mother can end up there and there is one midwife people gave her money, people encouraged her to deliver in the facility but nobody looked at the facility where she is going to deliver**** NLI006***

Similarly, respondents prioritised leadership and governance and felt they were important in ensuring that planned activities ran as planned. The limited decision and fiscal space at lower level MCH service provision were reported to impair the effective implementation of evidence-based interventions such as perinatal death surveillance and reviews. Where they are conducted, it always emerged that recommendations would sometimes not get implemented due to limited resources more so when they had to do with known chronic health systems challenges. This supportive working environment was equally seen as important to the health workers delivering these services.

*You have to improve all the aspects together. So, if it is leadership and governance you should improve that, you have to improve the financing, you have to improve service delivery, and human resources, you have to improve medical supplies and technologies and so all the aspects of health service delivery need to be improved**** NLI001***

Some respondents acknowledged the financial resource constraints and noted that widening the resource envelope, particularly that dispatched to health facilities as Primary Health Care (PHC) grants would address some of the challenges. The health worker numbers about the population served were highlighted as an important priority area. Current staffing norms at different levels of service provision were observed as not reflective of the gaps within the current service delivery. Some aspects of the policy did not empower midwives enough to address some of the emergency obstetric Care (EmOC) related cases and yet they form an important buffer which when addressed could reduce unnecessary referrals.

### Address the service user concerns

#### Quality concerns

Most respondents were open about their support for prioritisation of the demand side component which they felt needed to be attended to especially the quality of MCH care raised by the service users. They noted the need to prepare the health facilities to receive the mothers to address their expectations for quality service delivery. The provision of feedback about health and potential risks at every visit would maximise interaction with health workers. This may involve the promotion of service uptake and awareness creation regarding complimentary services.

*I think I remember also reading something on making sure you give feedback to the mother. That is one thing that we experienced when we implemented this project**** NLI003***

### Technical competencies

The focus on strengthening technical competencies was highlighted in positive regard and noted to have the potential to contribute immensely to current strategies. Entrenching skills to fully adhere to and implement evidence-based interventions would prove very beneficial. Equally important is the need to respond to the soft skills of health workers which would address attitude-related challenges. Enforcing guideline adherence was mentioned as a priority requiring specific focus. This moves in tandem with improving the skillsets of health workers who are handling this data. It emerged against the backdrop that the country had invested much in ensuring that all recommended practices were entrenched in the policy. However, the anticipated results are yet to be realised. This was reported to even be much better when subnational health systems are empowered enough to strengthen their data systems both at community and health facility levels. Improving medical supplies and commodities monitoring systems was identified as a priority for focus. It emerged that where systems were adequately strengthened, deficiencies in medicines and other supplies would render such investments ineffective.

*I think it is a work in progress. We are working with the Ministry of Health to see that some of the newborn and maternal health commodities are monitored efficiently across**** NLI008***

Stakeholders alluded to the need for repackaging postnatal care services to include content-sensitive and relevant mothers who experience a stillbirth. The inclusion of bereavement care was noted as equally important since its absence or minimal focus made stillbirth mothers leave the health facility without adequate preparation and knowledge of what could have happened in the first place and how to navigate health to avert risk and future occurrence. Supporting families through verbal autopsies to identify the cause was hoped to go a long way in turning communities into active participants in preventing community-level stillbirth risk factors.

*We shall go into another arm of notification which is the verbal autopsy. The biggest deaths of mothers are in communities and this system (DHIS2) cannot capture that but now with NIRA, we shall have a community arm through maybe the Village Health Teams (VHTs)**** NLI007***

### Community ownership

Opinions regarding enhancing community ownership of the current interventions were echoed by respondents. They observed that it was a viable strategy for the sustainability of interventions. They observed that common to most donor-supported interventions, aspects of community ownership were suboptimal. Community ownership for example of the referral efforts was particularly highlighted. The involvement of male partners would also supplement such efforts for better outcomes. Enhancing collaboration with community structures such as VHTs and other Civil Society Organisations (CSOs) was seen as an avenue for disseminating this information within the communities. Although part of the government strategy through community birth and death registration, the aspect of community notification of stillbirth had not gained root. This was partly because the birth and death registrations were still confined to the health facilities largely.

A policymaker acknowledged the need for political mobilisation and advocacy around stillbirth. It was observed that current efforts were minimal within the strategies, especially for those that impacted the communities directly. Unlike maternal mortality, stillbirth advocacy was reported to be minimal, especially in the rural areas where the problem is pronounced. It was reported to have benefits when patient advocates amplify their voices since they are the primary receivers of available services. Responding to cultural barriers, especially the negative ones around stillbirth was highlighted. Equally important was the need to share success stories such as the near miss, and recovery from adversity since current interventions have been implemented for some time.

### Continuum of care

Several policymakers recognised the need for adopting a continuum of care approach for stillbirth reduction which in many instances is already embedded in current MCH programming. They particularly mentioned it as a key strategy to respond to some of the risk factors which may lie outside pregnancy and childbirth. This could be possible through encouraging mothers and young girls to take up available Reproductive Maternal New-born and Child Health (RMNCH) services;

*We have to encourage the mothers right from; actually, the girls from the reproductive age to have first of all good nutrition to prepare them for birth, to prepare them for pregnancy because usually when the girls become pregnant before they mature they tend to have those stillbirths**** NLI012***

Repackaging ANC information session contents to reflect aspects of stillbirth risk factors and common causes of stillbirth was seen as important in creating awareness about stillbirth among mothers.

### Improve supervision

#### Regional structures

The need to strengthen the regional support supervision structures was highlighted as a priority going forward. Several stakeholders noted the need to strengthen regional structures to support MCH and avert stillbirth. Strengthening the management capacities of subnational-level MCH managers was identified as key. Supervision was particularly one aspect respondents mentioned as being key in improving strategies aimed at addressing the stillbirth burden. It was particularly highlighted that most projects that scored success were implemented with support from mentors based at the Ministry of Health headquarters. Whereas it was commendable due to the expertise they possessed, a suggestion was floated of strengthening the capacities of regional referral hospitals to be in a position to provide the same capacities. It was seen as a more sustainable solution given that such experts would be closer to the health workers and hence they can rely on them for technical backstopping. With improved support supervision, this was reported to yield better results which contributed to addressing the stillbirth burden in Uganda.

### Strengthen learning to inform practice

#### Popularise lesson sharing

Many informants thus emphasised the aspect of popularising lesson sharing as a national priority for stillbirth reduction strategies. Substantial learning had happened through the implementation of strategies for which some had produced good results. They observed the need to share such lessons to hold beneficial effects. The ability to learn from lessons so far was highlighted as an area requiring strengthening and further exploration. For example, much of the evidence that was used to inform the policies and intervention designs originated from piloted projects within and outside the country. The accruing learning thereof remains crucial and stakeholders observed that this needs to be standardised across the board and beyond individual pilot projects. A need for incentives to stimulate health workers to share their learning was raised. At a much broader level, the suggestion to establish learning hubs was shared. A need to strengthen information dissemination was seen as an avenue in ensuring that such information reached its intended beneficiaries.

Calls were made to entrench some of the lessons learnt from implementing these interventions into pre-service health worker training. A case emerged of the need to include aspects of perinatal death reviews into the training to empower health workers early on during the pre-service training;

*health worker training, pre-service, curricula, I think I have now the curricula has now been changed to put in a component of that so that you know there is a component of perinatal death review, pre-service training**** NLI001***

### Data ownership

Data ownership was also among the most commonly discussed aspects which required prioritisation. It was seen by stakeholders as an entry point into quality improvement efforts at the final point of service delivery. The promotion of data ownership was the first step towards securing commitment to improvement. The culture of data ownership at the health facility level was echoed. In particular, it was observed that despite generating some of the operational level data which could be crucial in addressing some of the implementation level bottlenecks, accepting such performance was a rare occurrence in some of the health facilities. Some data was generated purposely as routine to forward to subnational health managers but not to learn and improve service delivery from it. This disincentivised the practice thereby leading to its poor performance.

*What we have emphasized is that the health workers first own the data in terms of their performance in regards to saving babies because you can send these things to the district so that you can help the district but honestly as long as the end user who is this individual midwife with the individual mother and the baby is not empowered to critique their performance**** NLI006***

It felt realistic for program managers and implementers at the subnational level to use review meetings to explore avenues for obtaining lessons from current practice for better improvement of service delivery. They noted that these were already embedded in available policy provisions. However, respondents were sceptical about the motivation to hold such was context-specific, especially where previous resolutions were not implemented. The incentive to continue holding the same would dwindle. A proposal to strengthen such platforms was fronted in addition to strengthening the monitoring component within the MNCH care delivery.

*For infrastructure, we are monitoring the facilities that are being upgraded to make sure that they provide adequate services. In some of the programs that we have held, we have even worked to improve on the needed services in some of these facilities**** NLI018***

The promotion of operational research at the different health workers’ places of work was recommended as an avenue for identifying and responding to some of the operational-level challenges. Respondents noted that there weren’t many opportunities for funds to support such activities which made it an area for prioritisation to address the stillbirth burden;

*Although money for research is very little in terms of the country budgeting for research they do provide money for services**** NLI017***

### Opportunities

Despite highlighting priority areas of focus, there was consensus among stakeholders regarding the current opportunities which needed to be harnessed. They painted a brighter picture of the future upon modification of some of these aspects. They singled out various opportunities for improvement within the current strategies. A highlight of some follows below;

### Favourable policy environment

The favourable policy environment was raised as a potential opportunity for national strategies to address the stillbirth burden. Respondents noted that some of the current challenges experienced were not a result of the absence of policy provisions but rather inadequate application of policy. It was clear that operationalising the CEmONIC as stipulated in policy remained a challenge owing to other health systems’ bottlenecks. Ensuring uniform translation of policy provisions, especially in the hard-to-reach areas that bear the heaviest burden of stillbirth was observed as posing challenges. The presence of policy provisions was seen as a priority that could guide implementation in such areas and result in better performance. Currently, multiple opportunities for scaling up interventions exist within the health systems since most of the high-impact interventions have been integrated into policy and still could be adapted to the varying context. The increased focus on newborn health away from child health offers an opportunity to tailor efforts to the specific implementation needs of a newborn.

*There is a specific program because previously it would all be taken as child health together but there are now programs that are focused on newborn health**** NLI019***

### Harnessing the private sector contribution

Further, the potential role of the private sector especially in peri-urban and urban localities is linked to the possible areas for improvement in the national strategies to address the stillbirth burden. This is especially the case in peri-urban and urban areas where they concentrate. Bringing them on board in the national stillbirth response holds some benefits. Current statistics reflect a heavy burden of stillbirth in urban areas mostly arising from late referral and poor pregnancy and delivery management within private practitioners. However, their involvement in some of these quality improvement interventions was at the same time reported to be minimal like hands-on experience with the application of evidence-based tools. Harnessing their contribution through meaningful involvement was highlighted as a key priority for universal access to improved MCH services.

#### National level champions

There appeared to have emerged consensus among the respondents regarding the role of national-level champions. They observed that these had the potential to make a significant contribution by amplifying the current efforts to address the stillbirth burden. They noted that other aspects that may not have moved at the same pace could be raised by such actors using different platforms. A case in point was the training in emergency obstetric care, improving diagnostics capacity at different levels of MCH/EmOC service delivery among others. This they observed could be augmented with support to the patient advocates joining the efforts by amplifying the plight of the service users to highlight their experiences while interfacing with care.

### Consolidation of global support

Stakeholders recognised the potential of more benefits accruing from external financial and technical support. They observed that the onus was upon national-level actors to consolidate some of these efforts amidst increasing donor transition to central support. Harmonisation of guidelines from the different external sources and adapting them to the local context was one area. Currently, different projects are supporting various aspects of neonatal health, and harmonising implementation of such for better delivery was identified as a priority area requiring focus. The provision of quality reference points from global learning has helped support local efforts towards quality improvements. Uniform implementation of these efforts would go a long way in uplifting national standards. As many donor-supported projects are exiting, alignment and transfer of intervention to public health structures are gaining root. This is a priority contributing to health system strengthening. Varying aspects of continuous medical education from the partners such as clinical onsite mentoring could well be embedded within the routine standard of care. Besides, such efforts contribute to extra resources in terms of financing to support national-level implementation. This could further be enhanced by exploring the role and contribution of private philanthropists.

## Discussion

This study explored the stakeholders’ views regarding priorities and opportunities from the implementation of national stillbirth reduction strategies in Uganda. it presents valuable thoughts and insights from national stakeholders’ perspectives regarding how to better improve current strategies to address stillbirths. Respondents reflected on various issues which have been arranged into main themes that informed their views. While most stakeholders were aware of the national health systems context, their views shine a light on what can be achieved within this particular setting. An interpretation of each of the main findings follows below;

### Focus on all health systems components

The key finding from this study was the need to focus on all the health systems components particularly the supportive functions to enhance the efficiency at the health facility levels. A case in point was noted as the ongoing challenges with the referral systems. Perhaps this is reflective of the need to move away from project mode which was the anchor point when global stillbirth reduction campaigns were introduced in the country. As seen from the implementation elsewhere, intentional streamlining of the referral system saw a reduction in the first delay which minimised risks and in turn increased facility deliveries under skilled attendance [28, 29]. When not responded to, many mothers tend to report late which is reflected in the high numbers of fresh stillbirths which would have been avoided with available interventions timely. Indeed, the suboptimal facility deliveries under skilled attendance can partly be explained by this delay [30]. Challenges in accessing health facilities have been observed to contribute to the declining rates of ANC4 + despite a high turn-up for ANC1 [31, 32]. Elsewhere, it has also been found to contribute to the suboptimal utilisation of facilities for deliveries under a skilled attendant [33]. The inability to deliver under skilled attendance exposes mothers to higher risks of stillbirth [3, 8]. With the current implementation of strategies to address stillbirth already underway, it is important to dedicate efforts to the supportive functions of the health systems such as the streamlined referral system.

### Address the service user concerns

Findings further reveal the value stakeholders attached to the demand side component. Particularly responding to factors that would encourage and promote uptake of the available MCH services which would respond to stillbirth risk factors. The quality of MCH service concerns from the users affected their demand and utilisation of the same which affected community ownership of these interventions. This may have been so because as people were encouraged to take up available services, stillbirth started occurring within the health facilities which could have created doubt among service users about the quality of services. In addition, studies have demonstrated that many of the factors that continue to contribute to the invisibility of stillbirth originate from the negative community beliefs and practices around a stillborn [3, 33]. When community factors that draw away mothers from utilising available MCH services are addressed, it has the potential to increase visibility and has many mothers take up these services [34]. This avails the potential for timely identification of stillbirth risks and complications hence the health system’s ability for a timely response. The global campaigns highlighted community factors as one of the key intervention areas requiring stakeholders to devise interventions responding to the negative community beliefs and practices around stillbirth [2, 7]. When not responded to appropriately, many of the stillbirths happening in the community will continue going unnoticed [35]. Elsewhere, the main reason for underreporting the stillbirth data was the number of cases which go reported by the communities [36, 37] and the persistent misclassifications happening for facility-based stillbirths [38]. Attention to the demand side factors has the potential to improve the utilisation of available MCH services as seen from experiences elsewhere [3, 39]. It remains important for subnational level implementers therefore to embed the stillbirth prevention component within the ongoing demand side interventions of the MCH programs.

### Improve supervision

Results revealed that stakeholders placed a high priority on improvements within the supervision routines. Following capacity-building efforts for human resources, infrastructure and policy revisions in response to stillbirth reduction strategies, many stillbirth cases continue to occur within the health facilities. This is partly due to the inherent gaps in support supervision for the available MCH services. This may have been because some of the quality improvement functions are still at the centre which makes it difficult to implement in the absence of appropriate funding. Stringent support supervision has the potential for timely identification and response to identified gaps [40]. It can also facilitate collaborative work with health workers and managers to address the gaps and support the system to deliver quality MCH services [41]. Elsewhere, policy failures have been partly blamed on weak support supervision arrangements [42]. Strengthening supervision has been at the core of the MoH quality improvement strategies [43, 44]. When supervision is strengthened through mentorship, better MCH outcomes are realised [45] and more so when it is close to the implementation context. It remains paramount that supervision arrangements for available MCH services are further improved.

### Strengthen learning to inform practice

The study also revealed that the need to strengthen the learning component within the stillbirth reduction strategies was another priority area for focus among respondents. This may have been the observation given that policies have now been revised and rolled out, there may emerge a relapse among health systems managers to engage in the continuous process of learning to inform further revisions. Intentional and reflective learning from project implementation has the potential to unveil negative health systems responses to some of the introduced high-impact evidence-based interventions and tools [25, 46]. Early recognition of this can inform timely revision to align with the implementation context [47]. Delays in responding to negative health systems responses equally have the potential to derail intervention leading to the inability to achieve intended outcomes [48]. Elsewhere, contextual incompatibility with the rolled-out interventions has been partly blamed for the failure of pilots to thrive when scaled up into real-life health systems contexts [49, 50]. Strengthening learning, particularly from implementation level experiences by equipping health workers and managers with the necessary skills for data analysis and interpretation as well as implementation research skills will go a long way in improving interventions to fit the context and thereby responding to stillbirth risks [51, 52]. Most quality improvement frameworks are anchored on such theories of change from reflective learning.

### Favourable policy environment

The results also revealed that among the opportunities that stakeholders felt needed to be harnessed was the favourable policy environment. Policies provide clarity regarding implementation, and uniformity of the implemented interventions as well as a key reference point for guidance on implementation particularly in complex situations [2, 7]. The absence of guiding policy guidelines on the other hand creates ambiguity, subjecting practice to different interpretations depending on context and can lead to unintended consequences and adverse clinical outcomes [53]. The initial stages of the global stillbirth campaigns emphasized policy clarity highlighting it as a key intervention aspect to guide programming [2, 7]. It is therefore paramount that all efforts are made to ensure current MCH policy provisions are implemented as prescribed. By availing health workers with the required resources, it will aid in enforcing the policies in addition to the creation of a favourable implementation environment which supports the accurate execution of these policies.

### Harnessing the private sector contribution

Embracing the private sector’s contribution towards the national-level efforts to address the stillbirth burden was observed by the stakeholders as another opportunity worth emphasising. Results revealed that although the private sector contributed enormously towards MCH and particularly stillbirth reduction, their involvement in some national-level efforts remained minimal, especially the Private-for-profit (PFP) sector within the urban settings. This could have been the observation because current engagements tend to focus more on Private-not-for-profit (PNFP). Involvement of the PFP has the potential to respond to interlinkages particularly the referral aspects between the private to the public hence minimising unnecessary delays [54, 55]. The inability to meaningfully engage with the private sector may lead to continued delays and the practice of withholding mothers for far too long and yet facilities cannot manage the condition. A factor most likely the leading cause of urban MCH delays contributes to fresh stillbirths. Elsewhere, one of the avenues highlighted to drive the move towards Universal Health Coverage (UHC) was the meaningful involvement of the private sector to widen health service coverage [56, 57]. The same can be emulated in efforts aimed at increasing universal access to national strategies to address the stillbirth burden.

### National level champions

Lastly, the results highlighted another priority regarding the need for more engagement with national-level champions. This may have been the case since the role of the same actors towards the amplification of efforts to address the high maternal mortality is still fresh in respondents’ minds [1]. Engagement of such actors has the potential to bring to the fore, the need to rejuvenate efforts to address the national stillbirth burden [58]. The potential for relapse is one factor most common for such global health campaigns especially the duration following funding cessation [59]. Champions contribute to the maintenance of the country’s momentum towards a public health cause as was reflected in national efforts to address the high maternal mortality rates during the Millennium Development Goals (MDG) era [60]. Activation of this function would therefore call for deliberate efforts towards empowering patient advocates to add their voices on top of national level champions by equipping them with the necessary skills and offering them platforms to air out their concerns regarding service delivery.

### Limitation

This study is not without limitations; the findings were based on key informants’ opinions and experiences rather than empirical data on implementation effectiveness in the initial implementation of these evidence-based interventions into the health systems. Furthermore, key stakeholders in addressing the challenge of stillbirths are frontline health providers and birthing mothers, whose perspectives were not explored in the current study.

## Conclusion

In conclusion, therefore, great potential exists within the current strategies to address the national stillbirth burden. However, priorities such as improving the supportive functions of MCH service delivery and attention to the demand side need to be pursued for better service delivery with opportunities like a favourable policy environment to better serve the current strategies. This calls for dedicated efforts targeted at addressing gaps within the existing priorities and opportunities for better delivery of national strategies to address the stillbirth burden in Uganda.

### Electronic supplementary material

Below is the link to the electronic supplementary material.

Supplementary Material 1

## Data Availability

All data generated or analyzed during this study are included in this published article.

## Abbreviations

ANC: Antenatal Care
CEmONIC: Comprehensive Emergency Obstetric Neonatal Intensive Care
COREQ: Consolidated Criteria for Reporting Qualitative Research
CSOs: Civil Society Organisations
EmOC: Emergency Obstetric Care
HC: Health Centre
MCH: Maternal and Child Health
MDG: Millennium Development Goals
MoH: Ministry of Health
NGOs: Non-Governmental Organisation
PFP: Private-for-Profit
PNFP: Private-not-for-Profit
RMNCH: Reproductive Maternal New-born and Child Health
SRMNCH: Sexual and Reproductive Maternal New-born and Child Health
UHC: Universal Health Coverage
VHTs: Village Health Teams
