# Supplementary material for: Translating lessons to reinforce national stillbirth response; multi-stakeholder perspectives regarding priorities and opportunities to deliver quality evidence-based interventions within a limited-resource context in Uganda
Source: BMC Health Serv Res. 2024 Jun 10;24:715. doi: 10.1186/s12913-024-11180-z (PMC11165756; doi:10.1186/s12913-024-11180-z)
Supplement: Supplementary file 1 — Supplementary Material 1 [file 12913_2024_11180_MOESM1_ESM.docx]

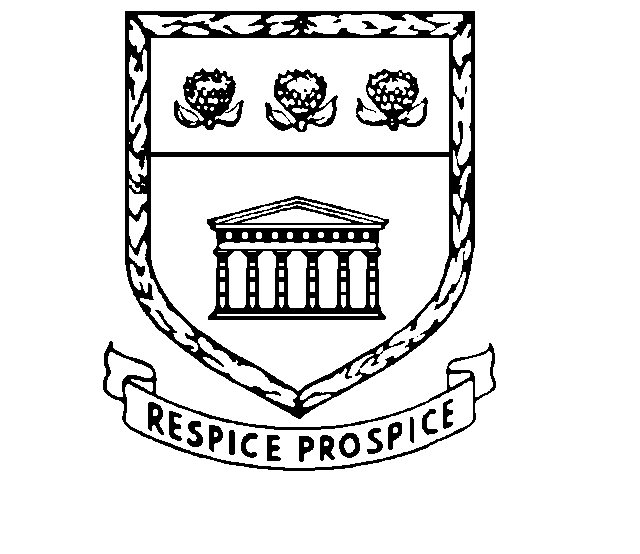


**
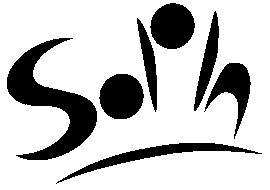
UNIVERSITY OF THE WESTERN CAPE**

**School of Public Health**

Private Bag X 17, Bellville 7535, South Africa

***Tel: +27 21-959 2809 Fax: 27 21-959 2872***

**E-mail:** [soph-comm@uwc.ac.za](mailto:soph-comm@uwc.ac.za)

**Title:** Risk Perceptions of Stillbirths among Women of Reproductive Age and Current Policy Response in Uganda; A case of Mukono and Buikwe districts

**Introduction**

This study is being conducted as part of a PhD in Public Health, as a requirement leading to a thesis for examination. The study will focus on your experiences with regards to how national Maternal Health Policies have been implemented to respond to the problem of still births in the country. If you agree to participate, completing the interview will take about [1 hour]. The interview is being administered by a research team member and will be provided to each key informant individually. The question guides in the tool include your understanding of the policy formulation and implementation processes, the influence of global and regional campaigns on the national maternal health direction, translation of policies to respond to stillbirths and the national level preparedness to address stillbirths. It is your decision whether or not to be in this study. You can stop your participation in the interview at any time or you can choose not to respond to specific questions in the tool but continue with the rest.

1. Would you kindly walk us through your current roles
2. In the policy arena what is being done to ensure that the number of stillbirth reduces
   1. When did you become aware that stillbirth is a major problem in Uganda
   2. What aspects of policy do you think currently addresses stillbirth
3. How have international guidelines been operationalized to ensure that they respond to the still birth problem locally?
   1. What has been the MoH role with regards to building capacities of districts to comply with these guidelines
4. Like for interventions such as helping baby breath and born too soon” is there anything of the sort that is looking at reducing still birth that occur around the time of delivery?
5. Over the years how have the maternal health policies policy making process evolved to incorporate aspects sensitive to addressing stillbirth since 2000.
6. What has been the MoH involvement of other stakeholders to ensure that current policies and guidelines are implemented
7. What efforts have been put in place to operationalize the every newborn action plan targets of 12/1000 stillbirths by 2030
8. What has been done by the ministry to ensure policies are implemented as per plan
9. What support has been provided to the districts and HSD to ensure that feedback on policy implementation is recorded and responded to
10. Overall how are policies being implemented in the country
11. How are other aspects like human resource, infrastructure, medicines and commodities aligned to ensure they support effective implementation of policies
12. Are there efforts to evaluate the failures of current policies to ensure they respond to still birth problem
13. What has been the effect of the global efforts to ensure Uganda picks attention to address stillbirth locally
14. How has the MOH worked to ensure that regional treaties with potential to address are implemented locally
15. What are you plans to ensure that stillbirth epidemic is addressed in future
